# 

**Published:** 1896-04

**Authors:** 


### CONTENTS:

### SELECTIONS.

Article I.—Harvard Odontological Society. - - - - 529

" II.—Surgery Without Pain. - - - - - - 539

" III.—Some Thoughts Touching Excess of Mineral or Calcareous Matter in the System. By John C. Story, M. D., D. D. S. - - - - - - 540

" IV.—Longevity. By F. H. Metcalf, D. D. S. - - 546

" V.—The Origin of Salivary Calculus. By Henry M. Burchard, D. D. S., M. D. - - - - - 554

" VI.—Anæsthesia as Produced by Schleich, of Berlin. By George Tully-Vaughan, M. D. - - - - 562

" VII.—An Apparatus for Chloroform Oxygen Anæsthesia. By Llewellys Eliot, M. D. - - - - - 565

### COMMUNICATED.

Illinois State Dental Society. - - - - - - - 568

### EDITORIAL, ETC.

Proposed Maryland Dental Law. - - - - - - 568

### MONTHLY SUMMARY.

Bicycling for Women. - - - - - - - - 570

Medical and Dental State Boards. - - - - - - 572

Hemorrhage. - - - - - - - - - 274

Hypnotism in Surgery. - - - - - - - - 574

New Method of General Narcosis. - - - - - - 575

Air-Chambers. - - - - - - - - - 575

"Doc." - - - - - - - - - - 576

As an Obtundant - - - - - - - - - 576

